# Production of OSU G5P[7] Porcine Rotavirus Expressing a Fluorescent Reporter via Reverse Genetics

**DOI:** 10.3390/v16030411

**Published:** 2024-03-07

**Authors:** Anthony J. Snyder, Chantal A. Agbemabiese, John T. Patton

**Affiliations:** 1 Department of Biology, Indiana University, 212 S. Hawthorne Drive, Simon Hall 011, Bloomington, IN 47405, USA; anthsnyd@indiana.edu (A.J.S.); cagbemabiese@noguchi.ug.edu.gh (C.A.A.); 2 Department of Electron Microscopy and Histopathology, Noguchi Memorial Institute for Medical Research, College of Health Sciences, University of Ghana, Accra 00233, Ghana

**Keywords:** rotavirus, reverse genetics, expression vector, porcine rotavirus

## Abstract

Rotaviruses are a significant cause of severe, potentially life-threatening gastroenteritis in infants and the young of many economically important animals. Although vaccines against porcine rotavirus exist, both live oral and inactivated, their effectiveness in preventing gastroenteritis is less than ideal. Thus, there is a need for the development of new generations of porcine rotavirus vaccines. The Ohio State University (OSU) rotavirus strain represents a *Rotavirus A* species with a G5P[7] genotype, the genotype most frequently associated with rotavirus disease in piglets. Using complete genome sequences that were determined via Nanopore sequencing, we developed a robust reverse genetics system enabling the recovery of recombinant (r)OSU rotavirus. Although rOSU grew to high titers (~10^7^ plaque-forming units/mL), its growth kinetics were modestly decreased in comparison to the laboratory-adapted OSU virus. The reverse genetics system was used to generate the rOSU rotavirus, which served as an expression vector for a foreign protein. Specifically, by engineering a fused NSP3-2A-UnaG open reading frame into the segment 7 RNA, we produced a genetically stable rOSU virus that expressed the fluorescent UnaG protein as a functional separate product. Together, these findings raise the possibility of producing improved live oral porcine rotavirus vaccines through reverse-genetics-based modification or combination porcine rotavirus vaccines that can express neutralizing antigens for other porcine enteric diseases.

## 1. Introduction

Reverse genetics systems have been developed for several *Rotavirus A* strains, including those that infect non-human primates (SA11 and RRV), humans (KU, CDC-9, HN126, Odelia, and RIX4414-like), cattle (RF), mice (rD6/2-2g), and birds (PO-13) [1,2,3,4,5,6,7,8,9,10]. These systems can be used for producing next-generation live oral vaccines, dual vaccine platforms that express foreign proteins, and diagnostic tools. Rotavirus (RV) accounts for a significant disease burden within important livestock, highlighting the need for effective animal RV vaccines. Nonetheless, no reverse genetics systems currently exist for any porcine RV, including those with the G5P[7] genotype, which represents the most frequent cause of disease in porcine populations [11,12].

RV is a major cause of acute gastroenteritis in piglets [11,12,13]. The virus is transmitted via the fecal–oral route and damages small intestinal enterocytes; as such, milk consumed by nursing piglets is not digested or absorbed into the intestines [12,13]. Moreover, RV is resistant to environmental factors, such as temperature, pH, and common disinfectants, which creates a persistent risk of infection [14,15,16]. Although RV-induced diarrhea is associated with low mortality and high morbidity, productivity losses create a significant economic burden on the global pork industry. Current vaccines only control diarrhea among infected populations [12,13]. Thus, a need exists for vaccines that are more effective.

Through modification of the RV genome though reverse genetics, the virus can be used as an expression vector of foreign proteins [1,2,3,17,18,19,20,21,22,23,24,25,26]. The RV genome is composed of 11 segments of double-stranded (ds)RNA. Each genome segment contains the coding sequence for a single protein except for segment 11, which expresses two proteins [27]. As one approach for using RV as an expression vector, the NSP3 open reading frame (ORF) in the segment 7 RNA is replaced with a modified ORF that encodes NSP3 fused to a foreign protein. In such modified segment 7 RNAs, the NSP3 stop codon is removed and a ~19 amino acid porcine teschovirus 2A translational stop-restart element is introduced between the coding sequences for NSP3 and the foreign protein. The 2A element (ATNFSLLKQAGDVEENPG/P) contains a canonical PGP (underlined) motif [28,29]. During translation of the 2A element, a peptide bond typically fails to form between the G and P residues of this motif. As a result, modified segment 7 RNAs generate two separate proteins: (i) NSP3 with 18 residual resides of the 2A element (NSP3-2A); (ii) a foreign protein that initiates with a P residue [2,3,19]. Modification of segment 7 RNAs in this manner has allowed for the generation of recombinant RVs that express fluorescent reporters, such as UnaG (green), mRuby (red), and TagBFP (blue), and other viral proteins, such as the norovirus VP1 capsid protein and the severe acute respiratory coronavirus-2 (SARS-CoV-2) S1 spike domain [2,3,17,18,19,20,21,22]. Notably, the segment 7 homolog of *Rotavirus C* strains encodes two separate proteins (NSP3 and dsRNA-binding protein) through the presence of an intervening naturally-occurring 2A element [30]. The modifications of RV segment 5 (NSP1) and 11 (NSP5) RNAs, in some cases involving the use of 2A elements, have revealed that recombinant *Rotavirus A* strains can express separate foreign proteins from at least three genome segments [23,24,25,26]. The ability of recombinant RVs to express foreign proteins may be useful in the generation of combination vaccines capable of inducing protective immune responses against RV and a second pathogenic virus.

We report a robust reverse genetics system for The Ohio State University (OSU) G5P[7] porcine RV. The sequences of its 11 dsRNA genome segments were determined via Nanopore sequencing of a laboratory-adapted OSU strain [31,32,33]. Using T7 expression plasmids designed using these sequences, we generated recombinant OSU (rOSU) and 11 rSA11/OSU monoreassortants. We also generated a rOSU isolate with a modified segment 7 RNA (rOSU-2A-UnaG) that expressed NSP3 and the UnaG reporter protein as separate products. Together, this work reveals (i) the first reverse genetics system for a porcine RV; (ii) a platform for making targeted genetic modifications of OSU; and (iii) a system that allows for the expression of foreign proteins during OSU infection. This system will enable detailed studies on the molecular mechanisms of porcine RV replication pathogenesis.

## 2. Materials and Methods

### 2.1. Cells and Virus

Embryonic monkey kidney (MA104) cells were grown in Dulbecco’s modified eagle medium (DMEM) containing 5% fetal bovine serum (FBS, Gibco) and 1% penicillin (10,000 U/mL)–streptomycin (10 mg/mL) (Quality Biological) at 37 °C in a 5% CO_2_ incubator [34]. Baby hamster kidney cells that constitutively express the T7 RNA polymerase (BHK-T7) were provided by Dr. Ulla Buchholz (Laboratory of Infectious Diseases, NIAID, NIH) and were grown in Glasgow minimum essential medium (GMEM) containing 5% heat-inactivated FBS, 1% penicillin–streptomycin, 2% 100X MEM-nonessential amino acids (NEAA) (Gibco), and 1% glutamine (200 mM) at 37 °C in a 5% CO_2_ incubator [35]. BHK-T7 cells were grown in medium supplemented with 2% Geneticin (50 mg/mL) (Gibco) in every other passage.

RVA/Pig-tc/USA/1975/OSU/G5P[7] was provided by Dr. Taka Hoshino (Laboratory of Infectious Diseases, NIAID, National Institutes if Health, Bethesda, MD, USA). This virus was activated by adjusting to a final concentration of 10 μg/mL porcine pancreatic trypsin, type IX (Millipore Sigma, Burlington, MA, USA), and incubating at 37 °C for 60 min. The activated virus was then propagated in MA104 cells maintained in serum-free DMEM with 0.5 μg/mL trypsin. The infected cells lysates were clarified via low-speed centrifugation at 1500× *g* for 15 min at 4 °C. Virus was isolated from the clarified lysates via extraction with an equal volume of Vertrel-XF (TMC Industries, Waconia, MN, USA) followed by ultracentrifugation at 100,000× *g* for 2 h at 4 °C. The pelleted virus [OSU-tc(MA104)] was resuspended in 500 μL of Tris-buffered saline and stored at −80 °C.

### 2.2. Nanopore Sequencing

OSU-tc_(MA104) dsRNA was extracted from 250 μL of clarified infected cell lysate using a Direct-zol RNA Miniprep Kit (Zymo Research, Irvine, CA, USA) following the manufacturer’s instructions. Prior to library preparation, the dsRNA was denatured with dimethylsulfoxide and poly(A)-tailed using New England Biolabs *Escherichia coli* Poly(A) polymerase following the manufacturer’s instructions. The poly(A)-tailed RNA was subjected to library preparation using an Oxford Nanopore Technologies (Oxford, UK) direct cDNA sequencing kit (SQK-DCS109) following the manufacturer’s instructions. The library was sequenced using an Oxford Nanopore Technologies MinION sequencer. Sequence assemblies of the 11 genome segments of OSU-tc_(MA104) were prepared using Geneious Primer software version 2023.2.1 [https://www.geneious.com/, accessed on 29 January 2024].

### 2.3. OSU Sequences Used in the Generation of T7 Expression Plasmids

The sequences of the OSU-tc_(MA104) genome segments were deposited in GenBank [https://www.ncbi.nlm.nih.gov/genbank/, accessed on 29 January 2024] under the accession numbers OP978238-OP978248. The sequences of modified segment 7 RNAs of OSU NSP3-2A (PP112343) and OSU NSP3-2A-UnaG (PP112344) were also deposited in GenBank.

### 2.4. Plasmids Used in This Study

Recombinant SA11 (rSA11) viruses were prepared using the plasmids pT7/SA11VP1, pT7/SA11VP2, pT7/SA11VP3, pT7/SA11VP4, pT7/SA11VP6, pT7/SA11VP7, pT7/SA11NSP1, pT7/SA11NSP2, pT7/SA11NSP3, pT7/SA11NSP4, and pT7/SA11NSP5 and pCMV/NP868R [1,3]. Recombinant OSU (rOSU) viruses were prepared using the plasmids pT7/OSUVP1, pT7/OSUVP2, pT7/OSUVP3, pT7/OSUVP4, pT7/OSUVP6, pT7/OSUVP7, pT7/OSUNSP1, pT7/OSUNSP2, pT7/OSUNSP3, pT7/OSUNSP4, and pT7/OSUNSP5, and pCMV/NP868R [3,35,36]. The pT7/OSU plasmids were made by Genewiz, Azenta Life Sciences (Waltham, MA, USA), based on OSU sequences determined via Nanopore sequencing. The plasmid pT7/SA11 NSP3-2A-UnaG was previously described [19]. The plasmids pT7/OSU NSP3-2A and pT7/OSU NSP3-2A-UnaG were produced by fusing DNA fragments for 2A or 2A-3xFLAG-UnaG, respectively, to the 3′-end of the OSU NSP3 open reading frame of pT7/OSU NSP3 using the In-Fusion cloning system (TaKaRa Bio, San Jose, CA, USA). Primer synthesis and plasmid sequencing were performed by EuroFins Scientific (Indianapolis, IN, USA) (Table 1).

### 2.5. Isolation, Amplification, and Analysis of Recombinant Viruses

RV reverse genetics was performed as previously described [3,35,37]. Briefly, BHK-T7 cells in 12-well plates were transfected with the 11 SA11 or OSU T7 plasmids, or combinations thereof, and with the capping enzyme plasmid, pCMV-NP868R, using Mirus TransIT-LT1 transfection reagent (Madison, WI, USA. Transfection mixtures contained 0.8 μg each of the pT7 plasmids, except for pT7/NSP2 and pT7/NSP5, which were used at 3-fold higher concentrations [2]. Two days post transfection, the BHK-T7 cells were overseeded with MA104 cells, and trypsin was added to the medium to a final concentration of 0.5 μg/mL. Three days later, the BHK-T7/MA104 cell mixtures were freeze–thawed thrice, and the lysates were clarified via low-speed centrifugation at 800× *g* for 5 min at 4 °C. To amplify the recovered viruses, the lysates were adjusted to 10 μg/mL trypsin and incubated for 1 h at 37 °C. MA104 cells in 6-well plates were then infected with 300 μL of the trypsin-treated lysates and incubated at 37 °C in a 5% CO_2_ incubator until all cells were lysed (typically 3–5 days). Recombinant viruses were recovered from the lysates via plaque isolation on MA104 cells [34,35]. Plaque-isolated viruses were initially grown on MA104 cells in 6-well plates and then, to generate larger pools, grown on MA104 cells in T175 tissue culture flasks at low multiplicity of infection (<1 plaque-forming unit [PFU]/cell). Briefly, 100 μL of the plaque-amplified lysates were activated via incubation with 10 μg/mL trypsin (final concentration). The activated lysates were diluted into 10 mL of serum-free DMEM and then used as inoculum to infect MA104 cells in T175 flasks. The flasks were placed at 37 °C in a 5% CO_2_ incubator for 1 h with rocking to ensure equal coverage of the inoculum over the monolayers. Following adsorption, the inoculum was removed and 25 mL of serum-free DMEM containing 0.5 μg/mL trypsin was added to each flask. The flasks were returned to the incubator until all cells were lysed (typically 3–5 days). The infected cell lysates were collected, clarified via low-speed centrifugation at 800× *g* for 5 min at 4 °C, and stored at −80 °C. Viral dsRNAs were recovered from the infected cell lysates via extraction with TRIzol (ThermoFisher Scientific, Waltham, MA, USA), resolved via electrophoresis on Novex 8% polyacrylamide gels (ThermFisher Scientific) in Tris-glycine buffer, detected via staining with ethidium bromide, and visualized using a Bio-Rad ChemiDoc MP imaging system (Hercules, CA, USA) [34,35,37]. Peak titers were determined from the infected cell lysates via plaque assay.

### 2.6. Plaque Assay

RV plaque assays were performed as previously described [34,35]. At 5 days post infection, MA104 monolayers with agarose overlays were incubated overnight with phosphate-buffered saline (PBS) containing 3.7% formaldehyde. The agarose overlays were then removed, and the monolayers were stained with 1% crystal violet in 5% ethanol for 3 h. The fixed and stained monolayers were rinsed with water and air dried. The plaque diameters were measured using ImageJ software [38]. Statistically significant differences in titer and plaque size were determined using ANOVA (GraphPad Prism, Boston, MA, USA).

### 2.7. Immunoblot Analysis

RV infections were performed as previously described [21,35]. Briefly, MA104 cells in 6-well plates were infected with 5 PFU/cell of the indicated viruses. At 9 h post infection, the infected cells were scraped into cold PBS. The infected cells were then washed with cold PBS, pelleted via centrifugation at 5000× *g* for 5 min at 4 °C, and lysed via incubation with nondenaturing lysis buffer (300 mM NaCl, 100 mM Tris-HCl [pH 7.4], 2% Triton X-100, and 1× EDTA-free Roche protease inhibitor cocktail [Sigma Aldrich, St. Louis, MO, USA]) for 30 min on ice. For immunoblot analysis, lysates were resolved via electrophoresis on 10% polyacrylamide gels in Tris-glycine buffer and transferred to nitrocellulose membranes [35,37]. After blocking with PBS containing 0.1% Tween-20 and 5% nonfat dry milk, the blots were probed with FLAG M2 antibody (F1804, Sigma Aldrich, 1:2000), 2A antibody (NBP2-59627, Novus, Centennial, CO, USA; 1:1000), RV VP6 antibody (lot 53963, 1:2000), or β-actin antibody (D6A8, Cell Signaling Technology [CST], Danvers, MA, USA; 1:2000). The bound primary antibodies were detected using 1:10,000 dilutions of horseradish peroxidase (HRP)-conjugated secondary antibodies (goat anti-mouse IgG [CST], goat anti-guinea pig IgG [KPL/SeraCare, Milford, MA, USA], or goat anti-rabbit IgG [CST]) in 5% nonfat dry milk. HRP signals were developed using the Bio-Rad Clarity Western ECL substrate and developed using a Bio-Rad ChemiDoc imaging system [35,37].

### 2.8. Genetic Stability Analysis

Genetic stability experiments were performed as previously described [3,18,19,20,21]. Briefly, the indicated viruses were serially passaged five times using 1:100 dilutions of infected cell lysates that were prepared in serum-free DMEM. When the cytopathic effect reached completion (typically 3–5 days), the cells were freeze–thawed thrice. Viral dsRNAs were recovered from the infected cell lysates via TRIzol extraction [35,37], resolved by electrophoresis on 8% polyacrylamide gels in Tris-glycine buffer, detected by staining with ethidium bromide, and visualized using a Bio-Rad ChemiDoc MP imaging system.

### 2.9. Assessment of Infectivity via Infectious Particle Production

RV infections were performed as previously described [21,35]. Briefly, MA104 cells in 6-well plates were infected with 5 PFU/cell of the indicated viruses. Following adsorption, the infected cells were washed thrice with PBS and incubated in serum-free DMEM containing 0.5 μg/mL trypsin at 37 °C in a 5% CO_2_ incubator. At the indicated times post infection, the infected cells were freeze–thawed thrice, clarified via low-speed centrifugation at 800× *g* for 5 min at 4 °C, and analyzed via plaque assay [34,35]. Statistically significant differences in titer were determined using ANOVA (Graph Pad Prism).

### 2.10. Assessment of Fluorescent Reporter Expression

RV infections were performed as previously described [21,35]. Briefly, MA104 cells in 6-well plates were infected with 0.05 PFU/cell of rOSU and rOSU-2A-UnaG. Following adsorption, the infected cells were washed thrice with PBS and incubated in serum-free DMEM containing 0.5 μg/mL trypsin at 37 °C in a IncuCyte S3 Live-Cell Analysis System (Sartorius, Bohemia, NY, USA). At 28 h post infection, images were acquired at 10× magnification under phase and green (excitation [440–480 nm], emission [504–544 nm]) channels.

### 2.11. Statistical Analyses

The results from all experiments represent three biological replicates. Horizontal bars indicate the means. Error bars indicate the standard deviations. *p*-values were calculated using one-way analysis of variance with Bonferroni correction (GraphPad Prism).

## 3. Results and Discussion

### 3.1. Recovery of OSU G5P[7] Porcine Rotavirus via Reverse Genetics

Genotype G5P[7] is representative of most RVs that cause acute gastroenteritis in suckling and weaned pigs, which leads to economic losses that plague the global pork industry [11,12]. Current treatments are generally ineffective at preventing disease [39,40,41,42,43]; thus, a need exists for robust molecular tools to develop next-generation porcine RV vaccines. Reverse genetics systems exist for several *Rotavirus A* strains [1,2,3,4,5,6,7,8,9,10]; however, no such system is available for a porcine RV. To address this knowledge gap, we utilized the well-studied Ohio State University (OSU) G5P[7] prototype strain [31,32,33]. Laboratory-adapted OSU (OSU-tc_(MA104)) was grown in MA104 cells, and viral dsRNA was extracted from infected cell lysates. The isolated RNA was then processed for Nanopore sequencing to obtain the complete sequences of all 11 genome segments (deposited in GenBank). Notably, the Nanopore sequences for 7 OSU-tc_(MA104) genome segments (VP2, VP3, VP6, VP7, NSP2, NSP3, and NSP5) were identical to those of the virulent (RVA/Pig-tc/USA/1975/OSU/G5P7/virulent) and attenuated (RVA/Pig-tc/USA/1975/OSU/G5P7/attenuated) strains. In contrast, VP1, VP4, and NSP1 were >99% identical, whereas NSP4 was >95% identical (nucleotide and amino acid sequence comparisons) [33]. The OSU-tc_(MA104) sequencing information was used to construct plasmids for reverse genetics experiments. Full-length cDNAs of each genome segment were positioned within T7 plasmids in between an upstream T7 RNA polymerase promoter and a downstream hepatitis delta virus ribozyme [35,37]. In the presence of the T7 RNA polymerase, the OSU T7 plasmids produce full-length, positive-sense RNA with authentic 5′ and 3′ termini.

**Figure 1:**
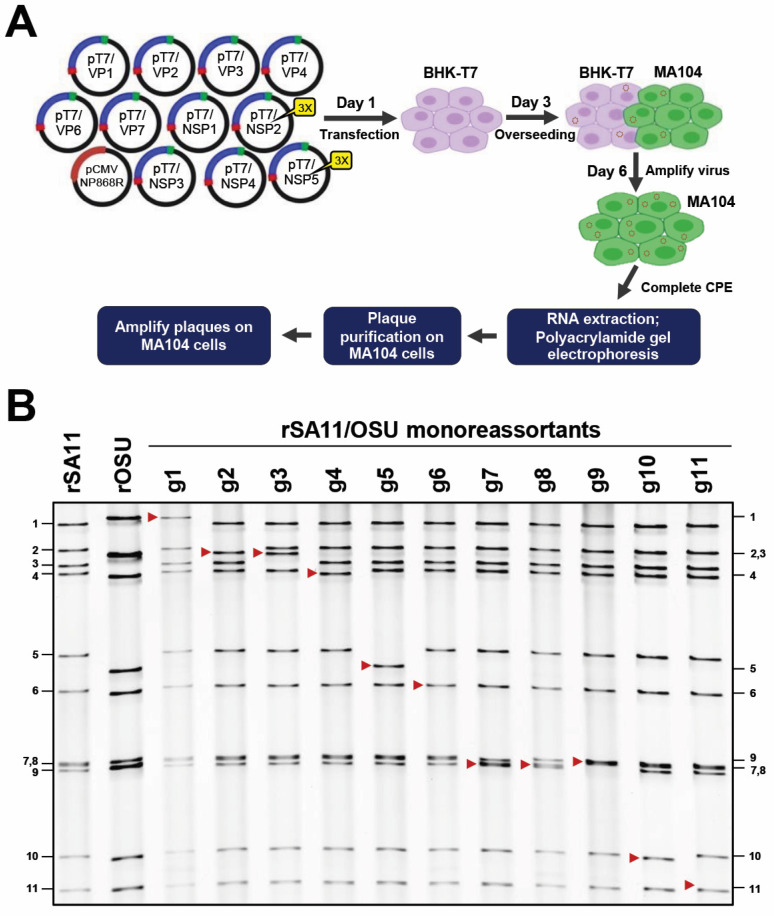
Production of OSU G5P[7] porcine rotavirus. (**A**) Rotavirus reverse genetics system [37]. Recombinant rotavirus was prepared by transfecting BHK-T7 cells with 11 T7 plasmids, which contain full-length cDNAs of rotavirus genome segments, and the CMV-NP868R plasmid, which encodes the African swine fever virus capping enzyme [3,36]. The BHK-T7 cells were overseeded 2 days post transfection with MA104 cells to facilitate the spread and amplification of recombinant rotavirus. At 3 days post overseeding, recombinant virus in the cells lysates was amplified on MA104 cells. The amplified virus was analyzed via RNA gel electrophoresis followed by plaque purification. Image was adapted from [37]. (**B**) Recovery of recombinant SA11/OSU monoreassortants via reverse genetics. Viral dsRNAs from rSA11, rOSU, and 11 rSA11/OSU monoreassortants were resolved via electrophoresis on an 8% polyacrylamide gel and stained with ethidium bromide. The migrations of rOSU gene segments in rSA11/OSU monoreassortants are indicated with red arrows. Genome segments 1–11 of rSA11 are indicated on the left side of the panel. Genome segments 1–11 of rOSU are indicated on the right side of the panel.

To confirm that each OSU T7 expression plasmid was functional, we generated 11 recombinant SA11/OSU (rSA11/OSU) monoreassortants. SA11 represents the prototype strain of simian RV [44,45,46]. Reverse genetics experiments were performed as previously described (Figure 1A) [35,36,37]. Briefly, 1 OSU T7 expression plasmid, 10 SA11 T7 expression plasmids, and pCMV-NP868R were transfected into BHK-T7 cells, which were subsequently overseeded with MA104 cells. Recombinant viruses generated in the transfected cells were amplified and their dsRNA profiles analyzed via gel electrophoresis. All transfection mixtures designed to produce monoreassortants resulted in the recovery of infectious virus (i.e., viral dsRNA) and induced a cytopathic effect within 5 days of infection. Thus, the genetic information obtained via Nanopore sequencing was functional for reverse genetics. By comparing the banding patterns to rSA11, we also determined the migration distance for each rOSU genome segment (Figure 1B, see red arrows). Functional differences between the SA11 and OSU genome segments and protein products may be investigated using the monoreassortants discussed here. Strikingly, we recovered recombinant virus that was composed of all 11 OSU genome segments (Figure 1B, see rOSU lane). Thus, we have developed a reverse genetics system for G5P[7] porcine RV that may be used for the production of vaccines targeting the most common cause of porcine RV infections. In contrast to current vaccines against porcine RV, which have been developed by serially passaging virulent strains in tissue culture, a costly and time-consuming process, the OSU reverse genetics system allows for the rapid generation of vaccine candidates and for the introduction of directed attenuating genetic mutations [41,42,47].

### 3.2. Recovery of OSU G5P[7] Porcine Rotavirus Encoding a Foreign Protein

**Figure 2:**
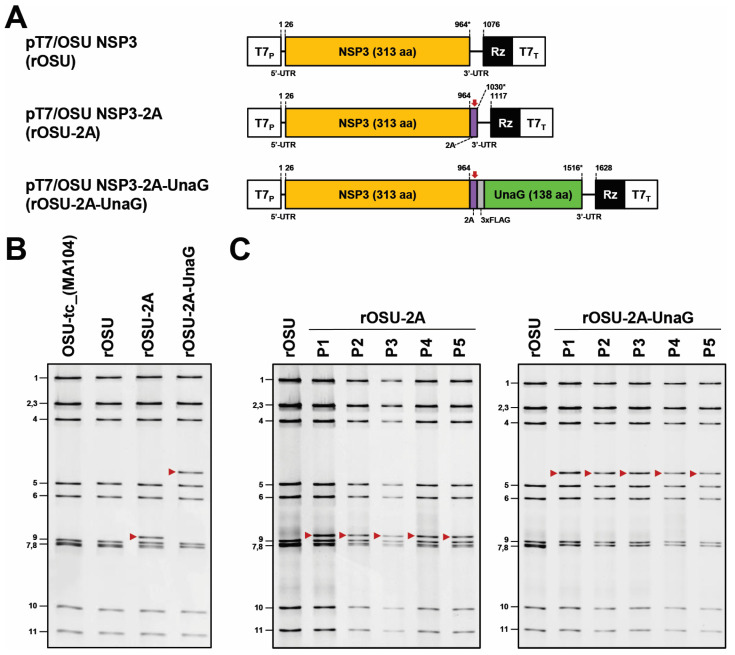
Production of recombinant OSU that encodes a foreign protein. (**A**) Modifications of rotavirus genome segment 7. The schematics indicate the nucleotide positions of the coding sequences for NSP3, the porcine teschovirus 2A element, 3X FLAG, and the fluorescent reporter UnaG (green). The red arrows indicate the positions of the 2A translational stop-restart elements, and the asterisks indicate the ends of the open reading frames. (**B**) Recovery of recombinant OSU-2A-UnaG via reverse genetics. Viral dsRNAs from OSU-tc_(MA104), rOSU, rOSU-2A, and rOSU-2A-UnaG were resolved via electrophoresis on an 8% polyacrylamide gel and stained with ethidium bromide. The migrations of modified genome segment 7 are indicated with red arrows. Genome segments 1–11 of rOSU are indicated on the left side of the panel. (**C**) Genetic stability. rOSU-2A and rOSU-2A-UnaG were serially passaged on MA104 cells. Viral dsRNAs from a total of five passages (P) were resolved via electrophoresis on an 8% polyacrylamide gel and stained with ethidium bromide. The migrations of modified genome segment 7 are indicated with a red arrow. Genome segments 1–11 of rOSU are indicated on the left side of the panels.

RV can be modified to express fluorescent reporters, such as UnaG (green), mRuby (red), and TagBFP (blue), and other viral proteins, such as norovirus VP1 and SARS-COV-2 S1 [1,2,3,17,18,19,20,21,22,23,24,25,26]). These recombinant viruses are valuable for analyzing RV biology via fluorescence-based imaging and may be used to induce protective immunity responses against multiple pathogenic viruses. As a proof of concept, we explored the possibility of expressing UnaG from OSU genome segment 7. The porcine teschovirus 2A-like (2A) element was positioned at the 3′ end of the NSP3 coding sequence, followed by the in-frame coding sequence of FLAG-tagged UnaG. Due to the activity of the 2A element [27,28,29], translation of the RNA produces two proteins: NSP3 with remnants of the 2A element and FLAG-tagged UnaG. We generated an additional expression plasmid with only the 2A element following NSP3 (Figure 2A). Using RV reverse genetics (Figure 1A), we recovered the rOSU-2A and rOSU-2A-UnaG viruses. For each, we observed a shift in the migration pattern of genome segment 7 to a larger molecular form (Figure 2B, see red arrows). These shifts are presumably due to the extra genetic material introduced into the segment 7 RNA by addition of the 2A and 2A-FLAG-UnaG coding sequences. We also noted similar dsRNA banding patterns for OSU-tc_(MA104) and rOSU, providing additional evidence for the utility of the OSU reverse genetics system.

To develop OSU G5P[7] porcine RV as a vector for expressing foreign protein, the recombinant virus must be genetically stable. This property is essential for scaling large quantities for vaccine production and for other applications. To examine genetic stability, we serially passaged rOSU-2A and rOSU-2A-UnaG at low multiplicity of infection. We observed no differences in the pattern of viral dsRNA recovered from the infected lysates during passage (Figure 2C, see red arrows). Notably, the 2A-FLAG-UnaG coding sequence is stable within the OSU background for at least five rounds of infection. This result is consistent with the genetic stability noted for other recombinant RV strains with modified segment 7 RNAs expressing UnaG (e.g., rSA11-2A-UnaG [19]). In contrast, insertion of foreign sequences longer that the 0.5-base UnaG sequence has been correlated with genetic instability, generating RV variants with segment 7 RNAs that retain the NSP3 ORF but lack all or portions of the foreign sequence [3,18,19,20,21]. Future studies will be required to determine if the introduction of longer foreign sequences into OSU segment 7 RNA likewise leads to instability.

### 3.3. Growth Characteristics of rOSU G5P[7] Rotaviruses

**Figure 3:**
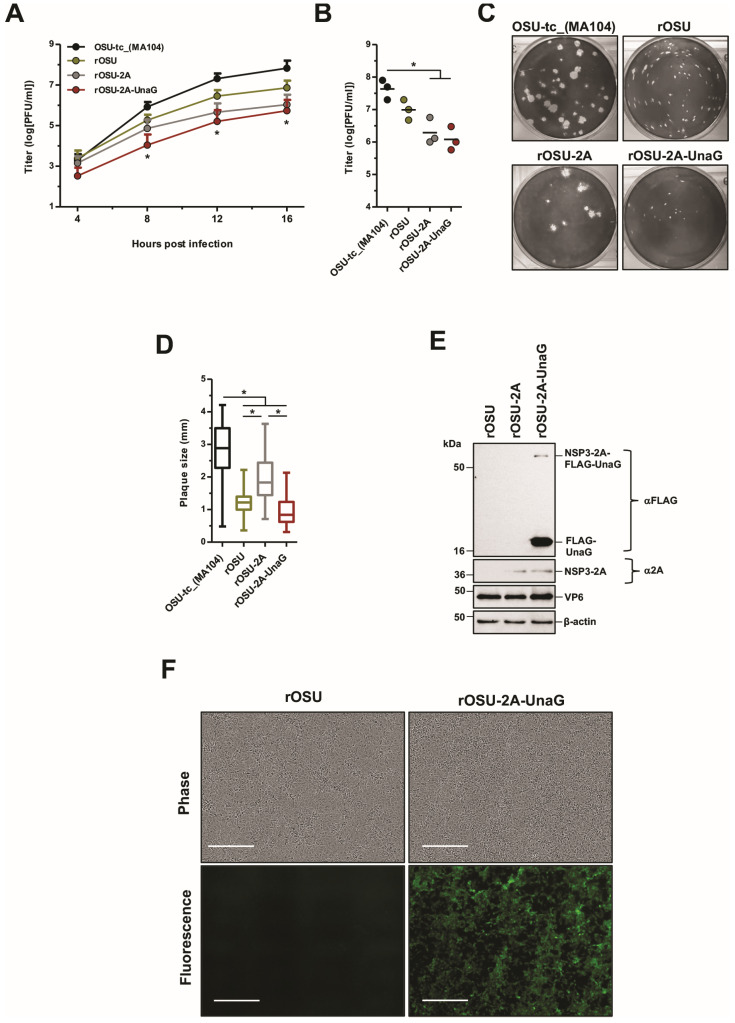
Characterization of recombinant OSU that expresses a foreign protein. (**A**,**B**) Production of infectious virus. MA104 cells were infected with the indicated viruses at an MOI of 5 PFU/cell. In panel (**A**), titers were determined via plaque assay at the indicated times post infection. In panel (**B**), titers were determined via plaque assay upon complete cytopathic effect (typically 3–5 days). Error bars indicate the standard deviations (**A**); horizontal bars indicate the means (**B**). In panel (**A**), statistical analyses show the comparisons between OSU-tc_(MA104) and rOSU, rOSU-2A, and rOSU-2A-UnaG; *, *p* < 0.05 (*n* = 3 biological replicate). (**C**,**D**) Plaque morphologies and sizes. In panel (**C**), plaques on MA104 cells were detected via crystal violet staining. In panel (**D**), plaque diameters were measured using ImageJ software [38]. Fifty plaques were measured for each virus; *, *p* < 0.05 (*n* = 3 biological replicates). (**E**,**F**) Fluorescent reporter expression and activity of the 2A translational stop-restart element. In panel (**E**), MA104 cells were infected with the indicated viruses at an MOI of 5 PFU/cell. At 9 h post infection, infected cell lysates were prepared and analyzed via immunoblot assay. FLAG-UnaG and the read through product, NSP3-2A-FLAG-UnaG, were detected using anti-FLAG antibody, and NSP3-2A was detected using anti-2A antibody. Positions of molecular weight markers are indicated on the left side of panels (*n* = three biological replicates). In panel (**F**), MA104 were infected with the indicated viruses at an MOI of 0.05 PFU/cell. At 28 h post infection, the infected cells were imaged at 10× magnification with an Incucyte live-cell analyzer (Satorius) using phase and green channels. Scale bars represent 400 µm (*n* = three biological replicates).

The recombinant viruses generated in this work were derived from sequencing information gained for the laboratory-adapted strain. To determine if these viruses exhibit similar growth characteristics, we compared their growth kinetics and plaque morphologies. The analysis showed that rOSU was a well-growing virus, reaching peak titers of ~10^7^ in MA104 cells. However, the peak titer reached by rOSU was ~0.5 log less than that reached by the OSU-tc_(MA104) virus. Moreover, single-step growth experiments indicated that rOSU grew slower than the OSU-tc_(MA104) virus. Plaque analysis also showed that the rOSU virus formed smaller plaques on MA104 cells than the OSU-tc_(MA104) virus (Figure 3A–D). These results suggest that sequence differences exist between rOSU and OSU-tc_(MA104) that impact virus growth. We conclude from this that the consensus sequence information generated for OSU-tc_(MA104) genome via Nanopore sequencing may not fully reflect the distribution and combinations of sequence variations in the OSU-tc_(MA104) population associated with the fittest, best-growing viruses. Indeed, the OSU-tc_(MA104) population likely represents a quasi-species, of which rOSU may or may not be a single variant. A recent study suggests that serial passage of clonal RV isolates (vis-à-vis plaque isolates) results in the introduction of mutations that favor increased growth kinetics [48]. In a similar vein, serial passage of the rOSU virus may lead to a change in its phenotype that more closely resembles the OSU-tc_(MA104) virus. Such experiments may reveal the nature of nucleotide and amino acid changes in the RV genome correlated with growth characteristics.

We next examined the growth characteristics of recombinant viruses that encode foreign protein. Compared to the rOSU, rOSU-2A-UnaG produced ~1 log-unit less infectious virus but generated similar plaque sizes (Figure 3A–D). This result is consistent with previous studies that show that the insertion of foreign genetic material into the segment 7 RNA is correlated with reduced virus growth [18,19,20,21].

### 3.4. Expression of Foreign Protein by rOSU G5P[7] Porcine Rotavirus

To develop OSU as a dual-vaccine platform, the vector must express a foreign protein. As such, the protein products made by rOSU-2A-UnaG in MA04-infected cells were probed via immunoblot assay using anti-FLAG antibody (Figure 3E). This assay revealed high levels of FLAG-UnaG (~18 kDa) expression, confirming that the rOSU-2A-UnaG virus directed expression of the UnaG foreign protein and contained a functional 2A element. We also detected minor amounts of the readthrough product, NSP3-2A-UnaG (~56 kDa); this likely derives from the failure of the 2A element to prevent peptide bond formation as the ribosome translates the PGP motif. Probing with an anti-2A antibody revealed a protein product that migrated at the expected molecular weight for NSP3 linked to the remnant residues of the 2A peptide (~38 kDa) (Figure 3E). Viral-protein VP6 and host-protein β-actin were detected under all infection conditions; however, NSP3-2A-UnaG, FLAG-UnaG, and NSP3-2A were not present in rOSU infected cell lysates. Finally, we used live cell imagining to determine whether the FLAG-UnaG expressed product of rOSU-2A-UnaG was functional. The results showed that in contrast to rOSU, rOSU-2A-UnaG produced high levels of fluorescent signal within live cells (Figure 3F). Thus, the rOSU-2A-UnaG virus expresses fluorescent UnaG protein, a feature not only enabling study of the replication and spread of the virus via live cell imagining but also suggesting that it may be possible to use recombinant OSU viruses as vectors in the development of combination vaccines.

## 4. Conclusions

The establishment of more effective porcine RV vaccines can be advanced through the application of reverse genetics technologies to create candidates that are superior in generating protective immunological responses. Such reverse genetics technologies allow for targeted genetic modifications and avoid the costly and time-consuming process of attenuating virulent strains through serially passage in vitro. Moreover, it may be possible to design combination vaccines that are capable of protecting individuals against RV and other pathogenic viruses. In this work, we developed a robust reverse genetics system for the G5P[7] OSU strain of porcine RV. Using information obtained by Nanopore sequencing the laboratory-adapted strain, we constructed 11 T7 expression plasmids that were sufficient for generating recombinant OSU (Figure 1). We leveraged the reverse genetics system to produce a recombinant virus that expressed UnaG within infected cells (Figure 2 and Figure 3). Recombinant viruses that express fluorescent reporters are valuable tools for monitoring virus spread in infected animals and assessing immunological responses in pigs.

The development of the porcine OSU reverse genetics system also has possible application for understanding the biology of human RVs, given the close similarity of the genotype constellations of the OSU virus and the human Wa-like genogroup RVs (e.g., G1P[8], G3P[8], and G12P[8] RVs) [49]. Notably, the genotype of the genome segments for the nonstructural proteins and the core structural proteins of OSU are generally the same as found for the Wa-like viruses: R1-C1-M1-A1-N1-T1-E1-H1. Indeed, phylogenetic analyses have indicated that the human Wa-like viruses evolved from OSU-like porcine RVs [50]. Unlike many animal viruses, the NSP1 protein of the OSU virus reportedly relies on a mechanism similar to that of the NSP1 proteins of the Wa-like viruses to antagonize the interferon signaling system [51]. As a result of the ease of growing porcine RVs and their genetic similarity to Wa-like viruses, the possibility of generating human RV vaccines from porcine virus strains has been explored [52].

## Figures and Tables

**Table 1:** Primers used in constructing OSU segment 7 expression platforms.

Constructed Plasmid	Template Plasmid for PCR	Primer	Sequence (5′ -> 3′)
pT7/OSU NSP3-2A	pT7/OSU NSP3	Destination Vector— Forward	TAGTCACATAATTTAAATATATTAA
		Destination Vector— Reverse	TAAATTATGTGACTAAGGACCGGGGTTTTCTTCCACGTCTCCTGCTTGCTTTAACAGAGAGAAGTTCGTTGCGCCGGCGCCTTCATATGTACATTCGTAGT
pT7/OSU NSP3-2A-UnaG	pT7/OSU NSP3-2A	Destination Vector— Forward	TAGTCACATAATTTAAATATATTAA
		Destination Vector— Reverse	TTCATATGTACATTCGTAGT
	pT7/SA11 NSP3-2A-UnaG	Insert—Forward	GAATGTACATATGAAGGCGCCGGCGCAACGAAC
		Insert—Reverse	TAAATTATGTGACTATTCTGTGGCCCTTCTGTAGCTC

## Data Availability

Data is contained within the article. Sequences used in the generation of recombinant OSU viruses are provided in NCBI GenBank {https://www.ncbi.nlm.nih.gov/genbank/, accessed on 29 January 2024} under accession numbers OP978238-OP978248, PP112343, and PP112344. Additional data related to this paper may be requested from the authors.
